# Variable Surface Glycoprotein from *Trypanosoma brucei* Undergoes Cleavage by Matrix Metalloproteinases: An in silico Approach

**DOI:** 10.3390/pathogens8040178

**Published:** 2019-10-08

**Authors:** Cláudia Jassica Gonçalves Moreno, Taffarel Torres, Marcelo Sousa Silva

**Affiliations:** 1 Programa de Pós-graduação em Bioquímica, Centro de Biociências, Universidade Federal do Rio Grande do Norte, Natal 59012-570, Brazil; claudia.mrn1@gmail.com; 2 Centro de Ciências Biológicas e da Saúde- Universidade Federal Rural de Semi-árido, Mossoró 59625-900, Brazil; taffarel.torres@ufersa.edu.br; 3 Departamento de Análises Clínicas e Toxicológicas, Centro de Ciências da Saúde, Universidade Federal do Rio Grande do Norte, Natal 59012-570, Brazil; 4 Global Health and Tropical Medicine, Instituto de Higiene e Medicina Tropical, Universidade Nova de Lisboa, 1800-166 Lisbon, Portugal

**Keywords:** variable surface glycoproteins, phospholipase C, matrix metalloproteinases, major surface protein, *Trypanosoma brucei*, African trypanosomiasis

## Abstract

In order to survive as extracellular parasites in the mammalian host environment, *Trypanosoma brucei* has developed efficient mechanisms of immune system evasion, which include the abundant expression of a variable surface glycoprotein (VSG) coat. VSGs are anchored in the parasite membrane by covalent C-terminal binding to glycosylphosphatidylinositol and may be periodically removed by a phospholipase C (PLC) and a major surface protein (TbMSP). VSG molecules show extraordinary antigenic diversity and a comparative analysis of protein sequences suggests that conserved elements may be a suitable target against African trypanosomiasis. However, the cleavage mechanisms of these molecules remain unclear. Moreover, in protozoan infections, including those caused by *Trypanosoma brucei*, it is possible to observe an increased expression of the matrix metalloproteinases (MMPs). To address the cleavage mechanism of VSGs, the PROSPER server was used for the identification of VSG sequence cleavage sites. After data compilation, it was observed that 64 VSG consensus sequences showed a high conservation of hydrophobic residues, such as valine (V), methionine (M), leucine (L) and isoleucine (I) in the fifth position—the exact location of the cleavage site. In addition, the PROSPER server identified conserved cleavage site portions of VSG proteins recognized by three matrix metalloproteases (gelatinases: MMP-2, MMP-3 and MMP-9). However, further biological studies are needed in order to analyze and confirm this prediction.

## 1. Introduction

Human African Trypanosomiasis (HAT) or sleeping sickness is a vector-borne disease caused by two hemoflagellate parasites named *Trypanosoma brucei gambiense* and *Trypanosoma brucei rhodesiense* [1]. Almost 60 million people are living in at risk areas in sub-Saharan Africa. There is no available vaccine and, therefore, rapid detection tests remain a priority for early treatment. Moreover, *Trypanosoma brucei brucei* parasites affect only animals—cattle and other ruminants [2]—causing important economic burden loss in endemic countries [3,4].

*T. brucei* have highly abundant variant surface glycoproteins (VSGs) expressed in the membrane, which provide numerous well-established functions on interaction with the mammalian host and even in the vector-*tsetse* flies’ life cycle [4]. VSG molecules from *T. brucei* are dimeric proteins that contain the same primary structure, composed of a combination of one N-terminal domain of approximately 340 residues and one or two C-terminal domains of 30 to 50 residues each. The C-terminal is attached to the plasma membrane by a glycosylphosphatidylinositol (GPI) anchor [5,6].

Briefly, VSG is a protective coat that is periodically changed to allow parasite manipulation and escape from the immune system [7,8,9,10,11]. Soluble VSGs released by *T. brucei* are extremely immunogenic molecules that cause a poor immune system response and progression to chronic infection [8,12,13]. Recently revised, the pattern of release of VSG molecules from the trypanosome surface is achieved by *T. brucei* endogenous phospholipase C (PLC) and major surface protein families (TbMSP A-C) [14]. A detailed analysis provided evidence that TbMSP and PLC expression is coordinately and inversely regulated during the differentiation of *T. brucei* parasites [13]. The PLC is only present in metacyclic and bloodstream stages of the life cycle. Nevertheless, loss of *T. brucei* plasma membrane integrity leads to GPI-PLC-mediated VSG hydrolysis [15,16]. Nevertheless, no definitive function for GPI-PLC has been identified.

However, GPI-PLC does not act alone in the VSG release process. TbMSP also participates in this VSG release mechanism during the antigenic variation mechanisms in *T. brucei*. TbMSP has homology with the major surface promastigote proteins from *Leishmania* species—etiological agents of leishmaniasis [17,18]. TbMSP is known to be a cell membrane-localized zinc metalloprotease expressed primarily in the process of the differentiation of the bloodstream form to the procyclic form of *T. brucei*. The TbMSP-B^−/−^PLC^−/−^ double mutant *T. brucei* line failed to differentiate into the procyclic form, with most VSGs remaining in the cell surface, unlike the single mutant protein. Thus, both TbMSP and PLC participate in the removal of the VSG coat during the differentiation of the bloodstream stage into procyclic trypanosomes [18]. Nevertheless, the dynamics of VSG coat replacement at the protein level remains unclear.

TbMSP is a zinc-dependent class of protein that shares similarities with the metzincins protein class, also known as matrix metalloproteinases (MMPs) [17,19,20]. It is believed that during infection by *T. brucei*, this protein could take part in extracellular matrix degradation, localization at the cell surface and proteolytic zinc-dependent activity [21,22,23,24]. Furthermore, the hydrolytic activities of TbMSP on proteins such as gelatin, casein, and matrix proteins such as collagen have been demonstrated [25]. Many studies showed an increased expression of MMP proteins during infection caused by trypanosomatids [26,27]. In addition, specific MMP inhibitors have been used to eliminate parasites as well as potential antiparasitic drugs [24,26,27,28].

In this work, we have investigated the relationship between MMP and VSG proteins due to the similarity of the proteolytic function of both MSPs and MMPs. Several VSG sequences have been deposited in the databank, as a result of extensively examined mechanisms regulating VSG gene diversification and expression [29,30]. VSG molecular organization resulted from a complex molecular process that involved many transcription processes and recombination to promote antigenic variation [31,32]. There are two domains of VSG proteins, a-VSG and b-VSG, that showed an evolutionary association, but with differences in the number and position of the conserved cysteine residue [33,34]. However, the a-VSG domain proved to be more diverse than the b-VSG domain [35].

Here, the National Center for Biotechnology Information (NCBI) databank was used to access and obtain the VSG sequences. All VSG sequences were submitted in the PROSPER server for the identification of possible endogenous cleavage sites and proteins that recognize these sequences.

## 2. Results

We deposited available VSG sequences from all subspecies present in the NCBI databank into the PROSPER server: *T. b. brucei*—Tbb (n = 236); *T. b. rhodesiense*—Tbr (n = 15) and *T. b. gambiense*—Tbg (n = 68). Overall, the analysis showed cleavage sites recognized for matrix metalloproteinases (MMPs) in all subspecies sequences and that they include the two VSG domains, A and B. After the sequence compilation, 46 consensus sequences (Table 1 and Table 2) were carried out. A high conservation of hydrophobic residues (black letters) was observed, such as valine (V), methionine (M), leucine (L) and isoleucine (I), specifically in the fifth position—the exact location of the cleavage site. Additionally, in Table 1, a high frequency of polar neutral residues can be observed next to the fifth position (green letters). The sequences were recognized in the first 100 amino acids conserved in ascending order by MMP9 > MMP3 > MMP2 (Table 1 and Table 2).

## 3. Discussion

This study underlines the importance of the in silico prediction of cleavage sites in all sequence regions of VSG molecules by matrix metalloproteinases (MMPs). The PROSPER server identified that all sequences have a conserved portion and specific amino acid cleavage site positions. Therefore, the activity of endogenous proteases would appear to be significant, essentially during microorganism infection. Previous studies, using a comparative analysis of different VSG sequences, suggest that a conserved element in this molecule could be an important target for an intervention strategy against African trypanosomiasis [6,10,36,37,38].

It has been demonstrated by Cross that the VSG coat could be uniformly removed from the cells by proteases, such as trypsin or pronase, without causing any changes in parasite morphology [39]. Consequently, maybe proteolytic activity could play a role in the mechanism of protein surface replacement during *T. brucei* infection. However, LaCount and colleagues described three classes of conserved families of zinc metalloproteases (TbMSP-A, -B and -C) present on *T. brucei* as responsible for the release of ectopically expressed VSG molecules from the surface of procyclic trypanosomes [8,17]. All of these three families of proteins are expressed in bloodstream-stage trypanosomes, but only TbMSP-B is found in the procyclic stage. The sequences of the three TbMSP share approximately 33% identity, and the main difference is in their terminals. The TbMSP-A has an extended C-terminal region, rich in serine and glutamate amino acid residues, which was not seen in the other two, and finishes in a short hydrophobic segment. TbMSP-B has a hydrophobic tail in the C-terminal. TbMSP-C has a C-terminal region that is highly hydrophilic and rich in charged amino acids and proline, which indicates that it is not linked to a membrane via a GPI anchor, unlike TbMSP-A and -B [17]. Moreover, the hydrolysis of PLC results in the conversion of the hydrophobic membrane form of VSG (mfVSG) to a water-soluble VSG (sVSG), resulting in the shedding of the VSG from the parasite membrane [40].

We observed hydrophobic sites on VSGs as substrates for, at the least, one matrix metalloproteinase, MMP-9 protein. Due to the higher number of recognized portions in all *T. brucei* subspecies sequences, matrix metalloproteinase proteins maybe able to release the VSG coat from the surface of the parasite. Additionally, VSGs molecules are available for interaction with other molecules besides MSPs and PL-C [19]. Our speculation is that MMPs may use VSG molecules as substrates in parasite–host interactions during *T. brucei* infection.

In physiological processes, MMPs regulate the release or activation of chemokines, cytokines, growth factors, and other bioactive molecules [24]. Since VSGs are antigenically distinct, the conserved structure is probably necessary for their function. It is possible that the high numbers of protease cleavage sites identified by the PROSPER server may be important for VSG release and antigenic variation during infection by *T. brucei.* Thus, the protein-conserved positions identified in cleavage sites might have a significant function and are accessible for surface cleavage by MMPs in different sizes.

Some questions remain unanswered. Can MPPs directly cleave VSGs to provide the release mechanism of VSG coats during the phenomenon of antigenic variation? Does increasing MPP expression during *T. brucei* infection represent an escape mechanism of the parasite or host protection?

To answer these questions, the data predicted by the PROSPER server was associated with the interaction between the parasite and mammalian host, described so far. Based on several studies, MMPs represent a large family of secreted or membrane-bound endopeptidases, important in many physiological and pathological conditions, including cancer and protozoan parasitic diseases [24,41]. MMPs play a crucial role in leukocyte penetration in brain diseases [39]. Consistently, mice infected with *T. brucei* have a high expression of MMP-3 and MMP-12 at the mRNA level, followed by significant parasitemia increases [24,28]. Apparently VSG proteins from *T. b. brucei* have many conserved sequences, including both domains A and B, that have undergone cleavage by MMPs (Table 1 and Table 2) and our results showed a significant number of cleavage sites recognized by MMP-3.

Besides, in the human infection context, some patients in the second stage of HAT (the neurological stage) present an increase in the expression of MMP-2 and MMP-9, correlated with the presence of parasites and leukocytes [20,24,42]. We could verify that MMP-2 and MMP-9 recognize several cleave sites of VSG domain A and B, present in *T. b. gambiense* and *T. b. rhodesiense* subspecies sequences. According to this finding, we believe that MMPs could be involved in VSG switch mechanisms and the release of soluble VSGs during infection caused by *T. brucei*. These data, together with the study model previously proposed by Moreno et al. [14], suggest that the mechanism of release of VSG in *T. brucei* may occur through the combination of host metalloproteinases and the intrinsic mechanisms of the parasite mediated by MSP and PLC enzymes.

Therefore, studies have shown that MMP inhibitors like antibiotics, such as tetracycline and minocycline, prolong the animal’s survival and decrease the influx of parasites and leukocytes in the brain [20,27,28]. Our recent studies focus on the evaluation of potential new drugs in the context of trypanosomatid infections. Thus, the mechanism of the antigenic variation of *T. brucei* seems to be an interesting target for the search for potential new antiparasitic molecules [43,44]. Consequently, understanding the mechanism of VSG release and all the involved molecules represents an important strategy for the development of new drugs against infections caused by *T. brucei*. In addition, a detailed analysis of all identified VSG conserved residues may provide new insights into host–parasite interactions, taking into account that released soluble VSGs interact directly with the immune system. Nevertheless, more studies are needed to validate these cleavage sites by MMPs.

## 4. Materials and Methods

The conserved domains for VSG sequences were retrieved from the Conserved Domain Database of the NCBI [45]. All *T. brucei* sequences were recovered from the NCBI protein database, then those sequences were divided considering the three subspecies: *T. b. brucei* (Tbb), *T. b. gambiense* (Tbg) and *T. b. rhodesiense* (Tbr). Data were sourced using the Reverse Position-Specific BLAST (RPS BLAST) tool v2.6.0 [46]. During the search, the NCBI CDD accessions cl03014 (a-VSG) and cl26244 (b-VSG) were used. In addition, the fragmented domains were discarded, and the minimum e-value threshold was 1.0 x 10^−5^.

**Figure 1:**
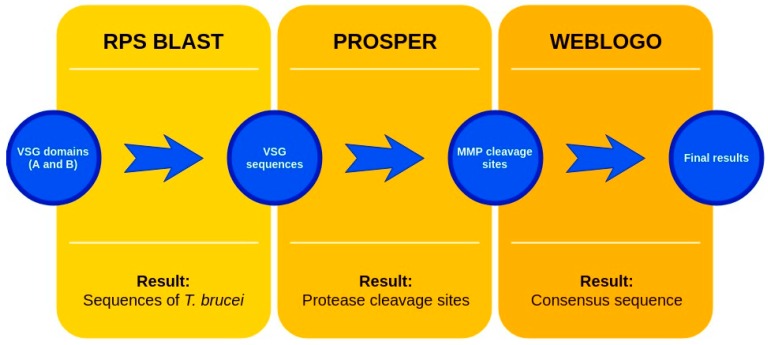
Methodologies overview: VSG domains A and B were analyzed by RPS BLAST after the VSG sequences were submitted to the PROSPER server to identify the cleavage sites. As a result, the protease cleavage sites were analyzed using the WebLogo tool to generate a consensus sequence.

The sequences that contain VSG domains were submitted to the PROSPER server to predict protease cleavage sites [46]. As an exclusivity criterion, the cleavage sites with a score greater than 0.8 were considered as probable sites for MMPs/MSPs. To demonstrate that the MMP/MSP sites present in *T. brucei* are very conserved, the standard sites for these proteases were retrieved from the MEROPS database [47] (Supplementary Materials Figures S1–S3). The cleavage sites were searched in all extensions of the protein sequence. To illustrate the similarity of the cleave sites, an analysis in the WebLogo tool was performed [48]. Our approach can be summarized as follows in Figure 1.

## 5. Conclusions

The antigenic variation mechanism is a sophisticated phenomenon that contributes to chronicity in the context of infections caused by *T. brucei*. Trypanosomes manipulate their hosts by VSG switch mechanisms as a strategy to successfully elude the immune system. However, here, using VSG sequence analysis, the matrix metalloproteinases (MMPs) were able to recognize several VSG sequences from different subspecies of *T. brucei.*

The interest in MMPs resulted from the observation that, besides their housekeeping role, they are involved in a wide range of physiological and pathological phenomena, including protozoa infections. Likewise, the VSG shedding mechanism by parasite protease is not fully understood. Therefore, we intended to analyze and identify VSG cleavage sites against protease present in the PROSPER server. Our study demonstrated that all the VSG sequences analyzed present conserved cleavage sites, which could be substrates for MMPs, such as MMP- 2, MMP-3 and MMP-9.

*T. brucei* present thousands of surface VSGs that could hypothetically interact with MMPs. Thus, the mammalian host may contribute in the infection dynamics through the overexpression of MMPs. Nevertheless, more biological studies are required to determine the functions of MMPs in the context of *T. brucei* infection.

## Figures and Tables

**Table 1:** Variable surface glycoprotein (VSG) domain A cleavage sites from *Trypanosoma brucei rhodesiense* (Tbr), *Trypanosoma brucei gambiense* (Tbg), and *Trypanosoma brucei brucei* (Tbb). Matrix metalloproteinases (MMP).

VSG Domain A (NCBI CDD * Accession: cl03014)		Number of MMP Cleavage Sites	MMP Cleavage Site Consensus Sequences Portion (WebLogo)
Tbb (85 sequences)	MMP2	309	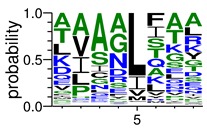
	MMP3	372	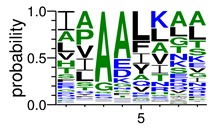
	MMP9	1673	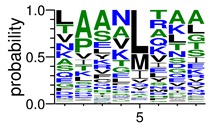
Tbr (5 sequences)	MMP2	17	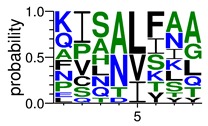
	MMP3	21	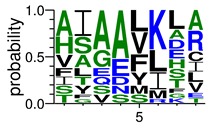
	MMP9	98	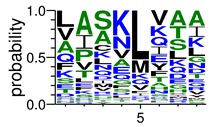
Tbg (22 sequences)	MMP2	58	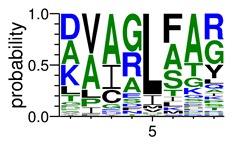
	MMP3	62	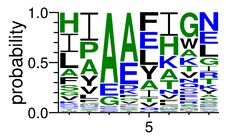
	MMP9	333	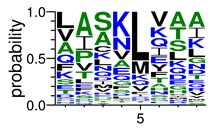

* CDD: Conserved Domain Database.

**Table 2:** Variable surface glycoprotein (VSG) domain B: cleavage sites from *Trypanosoma brucei rhodesiense (Tbr*), *Trypanosoma brucei gambiense* (Tbg), and *Trypanosoma brucei brucei* (Tbb).

VSG Domain B (NCBI CDD Accession: cl26244)		Number of MMP Cleavage Sites	MMP Cleavage Site Consensus Sequences (WebLogo)
Tbb (151 sequences)	MMP2	454	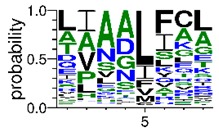
	MMP3	593	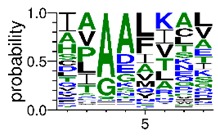
	MMP9	2983	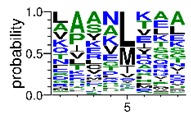
Tbr (10 sequences)	MMP2	17	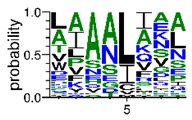
	MMP3	21	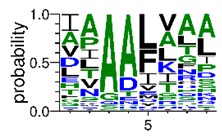
	MMP9	98	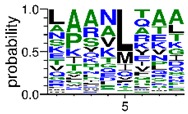
Tbg (49 sequences)	MMP2	145	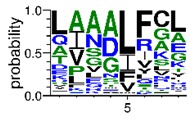
	MMP3	176	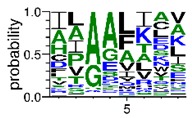
	MMP9	949	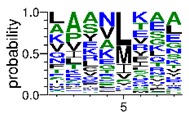

## Supplementary Materials

The following are available online at https://www.mdpi.com/2076-0817/8/4/178/s1, List S1: Used sequences (a few of sequences were used two times because were found in more than one subspecies of Trypanosoma brucei), Figure S1: Cleavege sites for matrix metallopeptidase-2 retrieved from MEROPS database, Figure S2: Cleavage sites for matrix metallopeptidase-9 retrieved from MEROPS database, Figure S3: Cleavage sites for matrix metallopeptidase-3 retrieved from MEROPS database.

## Abbreviations

HAT	Human African Trypanosomiasis
VSG	Variable surface protein
MSP	Major surface protein
PLC	Phospholipase C
MMPs	Matrix metalloproteinases
