# Supplementary material for: Variable Surface Glycoprotein from Trypanosoma brucei Undergoes Cleavage by Matrix Metalloproteinases: An in silico Approach
Source: Pathogens. 2019 Oct 8;8(4):178. doi: 10.3390/pathogens8040178 (PMC6963732; doi:10.3390/pathogens8040178)

pathogens

MDPI

List S1: Used sequences (a few of sequences were use two times because were found in more than one subspecies of Trypanosoma brucei).

1VSG (chain A and B), AAA30311.1, AAA57273.1, AAC72382.1, AAN78184.1, AAQ15663.1, AAQ16093.1, AAZ10285.1, AAZ10286.1, AAZ10919.1, AAZ10920.1, AAZ11134.1, AAZ11181.1, AAZ11614.1, AAZ11618.1, AAZ11694.1, AAZ11696.1, AAZ11698.1, AAZ11700.1, AAZ11702.1, AAZ12083.1, ABG66960.1, ABU50965.1, ABU50966.1, ACH41802.1, ACH41803.1, ACH41804.1, ACH41805.1, ACH41806.1, ACH41807.1, ACH41808.1 (two times), ACH41809.1, ACH41817.1, ACH41818.1, ACH41819.1, ACH41821.1, ACH41822.1, ACH41823.1, ACH41824.1, ACH41825.1, ACH41826.1, ACH41847.1, ACH41848.1, ACH41849.1, ACH41851.1, ACH41852.1, ACH41853.1, ACH41854.1, ACH41855.1, ACH41856.1, ACH41857.1, ACH41860.1, ACH41861.1, ACH41862.1, ACH41863.1, ACH41865.1, ACH41866.1, ACH41867.1, ACH41868.1, ACH41885.1, ACH41886.1, ACH41887.1, ACH41888.1, ACH41891.1, ACH41892.1, ACH41893.1, ACH41895.1, ACH41896.1, ACH41897.1, ACH41898.1, ACH41900.1, ACH41901.1, AEL79538.1, AEL79540.1, AEL79549.1, AEL79559.1, AEL79568.1, AGG79442.1, AGG79443.1, AGG79445.1, AGG79446.1, AGG79447.1, AGG79448.1, AGG79462.1, AGG79463.1, AGQ50299.1, AGQ50300.1, AHW98113.1, CAC33792.1, CAC33895.1, CAC87890.1, CAC87891.1, CAD90550.1, CAI77634.1 (two times), CAI77635.1, CAI77636.1, CAI77637.1, CAI77638.1, CAI77639.1, CAI77640.1, CAI77644.1, CAI77645.1, CAI77646.1, CAI77647.1, CAI77649.1, CAJ15942.1, CAJ16160.1, CAJ16171.1, CAJ16199.1, CAJ16204.1, CAJ16209.1, CAJ16211.1, CAJ16243.1, CAJ16269.1, CAJ16282.1, CAJ16290.1, CAJ16308.1, CAJ16345.1, CAJ16395.1, CAJ16467.1, CAJ16478.1, CAJ16695.1, CAJ16712.1, CAJ16716.1, CAJ16723.1, CAJ16737.1, CAJ16740.1, CAJ16767.1, CAJ16769.1, CAJ16800.1, CAJ16807.1, CAJ16811.1, CAJ16828.1, CAJ16845.1, CAJ16846.1, CAJ16853.1, CAJ16856.1, CAJ16863.1, CAJ16874.1, CAJ16889.1, CAJ16900.1, CAJ16911.1, CAJ16920.1, CAJ16974.1, CAJ16992.1, CAJ17037.1, CAJ17081.1, CAJ17124.1, CAJ87066.1, CAQ55501.1, CAQ57305.1, CAQ57308.1, CAQ57316.1, CAQ57328.1, CAQ57340.1, CAQ57355.1, CAQ57368.1, CAQ57400.1, CAQ57406.1, CAQ57415.1, CAQ57423.1, CAQ57439.1, CAQ57445.1, CAQ57460.1, CAQ57474.1, CAQ57486.1, CBG92828.1, CBG92829.1, CBG92832.1, CBG92833.1, CBG92834.1, CBG92836.1, CBH08934.1, CBH09346.1, EAN76223.1, EAN76225.1, EAN76228.1, EAN76245.1, EAN76250.1, EAN76254.1, EAN76257.1, EAN76754.1, EAN76757.1, EAN76761.1, EAN76762.1, EAN76766.1, EAN77377.1, EAN77379.1, EAN77382.1, EAN77385.1, EAN77413.1, EAN77417.1, EAN77418.1, EAN77420.1, EAN77424.1, EAN77425.1, EAN77428.1, EAN77430.1, EAN77439.1, EAN78980.1, EAN78981.1, EAN78982.1, EAN78985.1, EAN78986.1, EAN78989.1, EAN78990.1, EAN78991.1, EAN78994.1, EAN79375.1, EAN80116.1, EAN80406.1, EAN80407.1, EAN80650.1, EAN80651.1, EAN80654.1, EAN80655.1, EAN80656.1, EAN80660.1, EAN80663.1, EAN80664.1, EAN80669.1, EAN80670.1, EAN80672.1, EAN80673.1, EAN80676.1, EAN80678.1, EAN80679.1, EAN80680.1, EAN80681.1, EAN80682.1, EAN80683.1, EAN80684.1, EAN80685.1, P02896.1, P02897.1, P02898.1, P06013.1, P06014.1, P06015.1, P06016.1, P20946.1, P20947.1, P21840.1, P26326.1, P26327.1, P26328.1, P26329.1, P26330.1, P26331.1, P26332.1, P26334.1, XP_011771375.1, XP_011771652.1, XP_011771964.1, XP_011771967.1, XP_011771970.1, XP_011772076.1, XP_011772206.1, XP_011772207.1, XP_011772208.1, XP_011772917.1, XP_011773158.1, XP_011773239.1, XP_011773700.1, XP_011773770.1, XP_011773822.1, XP_011773910.1, XP_011774306.1, XP_011775153.1, XP_011775154.1, XP_011775156.1, XP_011775157.1, XP_011775158.1, XP_011775328.1, XP_011775329.1, XP_011776258.1, XP_011776260.1, XP_011776263.1, XP_011776264.1, XP_011776593.1, XP_011777172.1, XP_011780452.1, XP_011780564.1, XP_011780782.1, XP_011780783.1, XP_843642.1, XP_843645.1, XP_843646.1, XP_843647.1, XP_843648.1, XP_843744.1, XP_843745.1, XP_843746.1, XP_843847.1, XP_844689.1, XP_844691.1, XP_844692.1, XP_844693.1, XP_844694.1, XP_844697.1, XP_844699.1, XP_844703.1, XP_845069.1, XP_845136.1, XP_845138.1, XP_845139.1, XP_845141.1, XP_845142.1, XP_845143.1, XP_845634.1, XP_845635.1, XP_845637.1, XP_846273.1, XP_846274.1, XP_846883.1, XP_846884.1, XP_846885.1, XP_847559.1, XP_847561.1

Figure 1. Cleavage sites for matrix metallopeptidase-2 retrieved from MEROPS database (https://www.ebi.ac.uk/merops/cgi-bin/pepsum?id=M10.003).

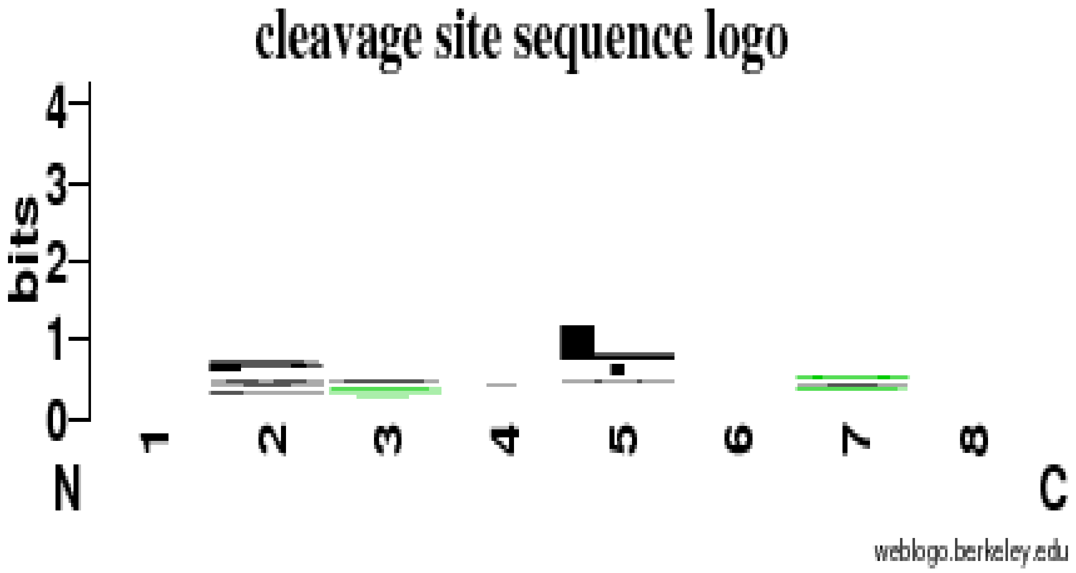

Figure 2. Cleavage sites for matrix metallopeptidase-9 retrieved from MEROPS database (https://www.ebi.ac.uk/merops/cgi-bin/pepsum?id=M10.004).

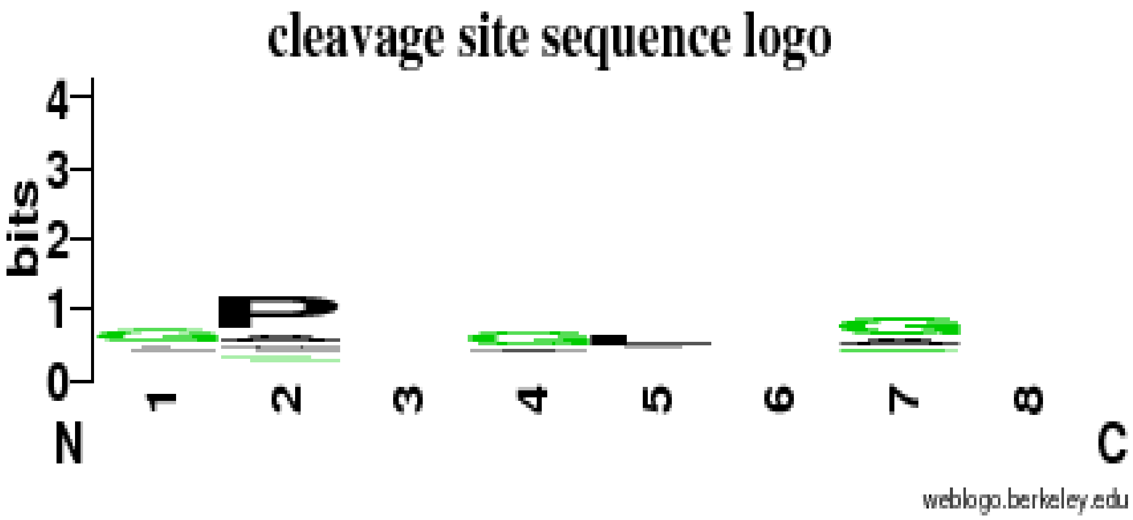

Figure 3. Cleavage sites for matrix metallopeptidase-3 retrieved from MEROPS database (https://www.ebi.ac.uk/merops/cgi-bin/pepsum?id=M10.005).

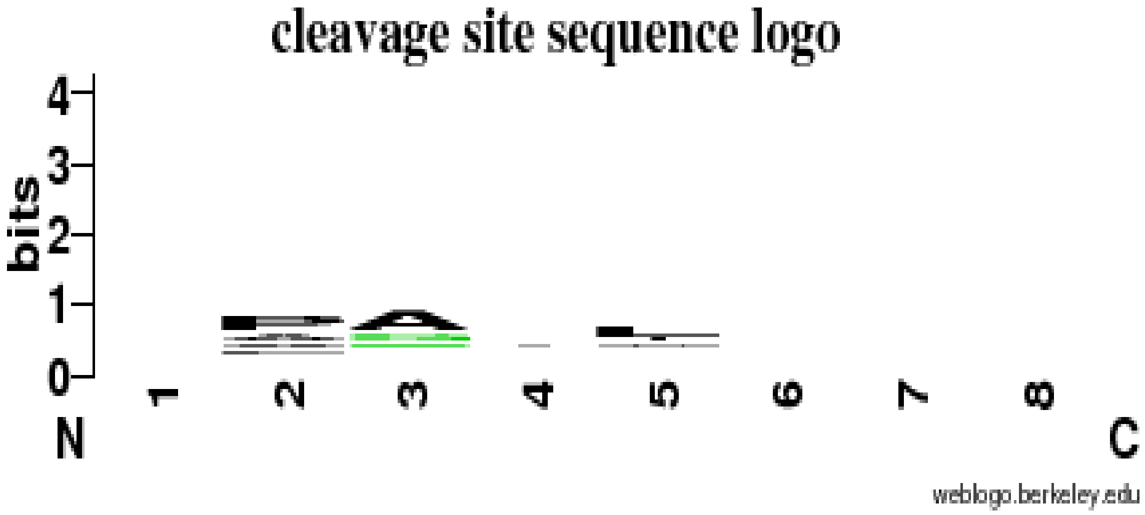

Supplement: Supplementary file 1 [file pathogens-08-00178-s001.pdf]
